# MR-Class: A Python Tool for Brain MR Image Classification Utilizing One-vs-All DCNNs to Deal with the Open-Set Recognition Problem

**DOI:** 10.3390/cancers15061820

**Published:** 2023-03-17

**Authors:** Patrick Salome, Francesco Sforazzini, Gianluca Grugnara, Andreas Kudak, Matthias Dostal, Christel Herold-Mende, Sabine Heiland, Jürgen Debus, Amir Abdollahi, Maximilian Knoll

**Affiliations:** 1 Clinical Cooperation Unit Radiation Oncology, German Cancer Research Center, 69120 Heidelberg, Germany; 2 Heidelberg Medical Faculty, Heidelberg University, 69117 Heidelberg, Germany; 3 German Cancer Consortium Core Center Heidelberg, 69120 Heidelberg, Germany; 4 Heidelberg Ion-Beam Therapy Center, 69120 Heidelberg, Germany; 5 Department of Neuroradiology, Heidelberg University Hospital, 69120 Heidelberg, Germany; 6 Department of Radiation Oncology, Heidelberg University Hospital, 69120 Heidelberg, Germany; 7 Clinical Cooperation Unit Radiation Therapy, German Cancer Research Center, 69120 Heidelberg, Germany; 8 Brain Tumour Group, European Organization for Research and Treatment of Cancer, 1200 Brussels, Belgium; 9 Division of Neurosurgical Research, Department of Neurosurgery, University of Heidelberg, 69117 Heidelberg, Germany

**Keywords:** content-based image classification, data curation and preparation, convolutional neural networks (CNN), deep learning, artificial intelligence (AI)

## Abstract

**Simple Summary:**

MR-Class is a deep learning-based MR image classification tool for brain images that facilitates and speeds up the initialization of big data MR-based studies by providing fast, robust and quality-assured imaging sequence classifications. Our studies observed up to 10% misclassification rates due to corrupt and misleading DICOM metadata. This highlights the need for a tool such as MR-Class to help with data curation. MR-Class can be integrated into workflows for DICOM inconsistency checks and flagging or completing missing DICOM metadata and thus contribute to the faster deployment of clinical artificial intelligence applications.

**Abstract:**

Background: MR image classification in datasets collected from multiple sources is complicated by inconsistent and missing DICOM metadata. Therefore, we aimed to establish a method for the efficient automatic classification of MR brain sequences. Methods: Deep convolutional neural networks (DCNN) were trained as one-vs-all classifiers to differentiate between six classes: T1 weighted (w), contrast-enhanced T1w, T2w, T2w-FLAIR, ADC, and SWI. Each classifier yields a probability, allowing threshold-based and relative probability assignment while excluding images with low probability (label: unknown, open-set recognition problem). Data from three high-grade glioma (HGG) cohorts was assessed; C1 (320 patients, 20,101 MRI images) was used for training, while C2 (197, 11,333) and C3 (256, 3522) were for testing. Two raters manually checked images through an interactive labeling tool. Finally, MR-Class’ added value was evaluated via radiomics model performance for progression-free survival (PFS) prediction in C2, utilizing the concordance index (C-I). Results: Approximately 10% of annotation errors were observed in each cohort between the DICOM series descriptions and the derived labels. MR-Class accuracy was 96.7% [95% Cl: 95.8, 97.3] for C2 and 94.4% [93.6, 96.1] for C3. A total of 620 images were misclassified; manual assessment of those frequently showed motion artifacts or alterations of anatomy by large tumors. Implementation of MR-Class increased the PFS model C-I by 14.6% on average, compared to a model trained without MR-Class. Conclusions: We provide a DCNN-based method for the sequence classification of brain MR images and demonstrate its usability in two independent HGG datasets.

## 1. Introduction

Magnetic resonance imaging (MRI) has become a crucial imaging modality in the detection and staging of various types of cancer, including brain tumors [1]. With its high-resolution images, MRI can distinguish between healthy and diseased tissues, providing high soft-tissue contrast and overcoming limitations of other imaging techniques, specifically in disease monitoring [1]. Therefore, there has been an increase in interest in MRI-based artificial intelligence (AI) applications and studies in recent years [2]. An essential step in the data preparation phase of such studies is accurately classifying MR images, since each image communicates specific anatomical or physiological information [1]. An example is brain tumor segmentation algorithms requiring information from multiple MR modalities, as distinguishing between healthy brain tissue and tumors is often challenging. Ensuring that the right sequences are used for analysis, i.e., the classification of sequences, can be a demanding and time-intensive task. This is particularly true when dealing with a large amount of data from different sources, such as multiple scanners and treatment centers, which may have inconsistent naming schemes. In particular, retrospective data collection introduces additional challenges (non-prespecified protocols and sequences).

Gueld et al. found that relying on image metadata (i.e., information stored in the DICOM header) to classify medical images can often lead to unreliable results [3]. This is because there may not always be a consistent match between the DICOM tags and the actual examination protocols used. These inconsistencies are often introduced in order to improve the quality of the imaging, such as implementing different imaging protocols for different regions of the body to account for variabilities in patients’ anatomies [3]. Harvey et al. report data labeling as the costliest part of radiomics studies [4] and that consistent and unbiased labeling should be performed across the entire dataset to yield robust machine learning models [4]. However, this can be challenging when large amounts of data are considered. Therefore, automating the retrieval of medical images and classifying data based on its content would bring several advantages, including improved time efficiency, accuracy, and ultimately, reproducibility.

Compared to text-based image classification, content-based image classification (CBIC) is independent of inconsistencies between different image sources, is unaffected by human error, and is less labor-intensive [5]. CBIC methods for medical images include the use of traditional classification machine learning techniques such as K-nearest neighbor (kNN) [6], support vector machine [7] (SVM), as well as deep learning methods [8]. After the success of the deep convolutional neural network (DCNN) AlexNet [9] in the ImageNet [10] classification challenge, an increase in interest in DCNN has been seen when dealing with image classification tasks [11,12,13]. Regarding the utilization of DCNNs for medical image retrieval and classification, four studies have been identified that achieved high levels of accuracy (>90%) in classifying body organs and MR images [14,15,16,17]. A summary of these models can be seen in Supplementary Table S1. One limitation of these methods is their inability to address the open-set recognition problem, which refers to the failure of a network trained to classify a specific number of classes to handle unknown classes [18]. This problem is frequently encountered in clinical cohorts since datasets exported from hospitals’ picture archiving and communication systems (PACS) usually comprise all available medical images and data, leading to various medical image modalities and sequences.

This work addresses the open-set recognition problem by training a DCNN-based MR image classifier using a one-vs-all approach. Following a comparison study of published DCNNs for medical image classification to determine the chosen DCNN model, one-vs-all binary class-specific DCNN classifiers were trained to recognize a particular MR image, thus forming MR-Class.

## 2. Materials and Methods

### 2.1. Datasets

This study included three datasets: The training/validation cohort (C1) consisted of 320 primary/recurrent high-grade glioma (HGG) patients with a median of 9 image acquisition time points, resulting in 20,101 MR images acquired between 2006 and 2018. The dataset was collected retrospectively from 23 scanners at the Heidelberg University Hospital (UKHD). The first testing cohort (C2) consisted of 197 HGG patients, with a median of 7 time points, resulting in 11,333 images acquired between 2009 and 2017. The dataset was collected retrospectively from 15 different scanners at the UKHD. A public data cohort (C3) was also utilized for the second testing of MR-Class. The data cohort was retrieved from the Cancer Genome Atlas Glioblastoma Multiforme (TCGA-GBM) data collection [19]. The cohort included scans from 256 GBM patients with a median of 3 time points, resulting in 3522 MR images acquired between 1986 and 2019 and collected from 17 scanners. Patient demographics of all three cohorts can be seen in Supplementary Table S2.

### 2.2. MR Scans

**Figure 1:**
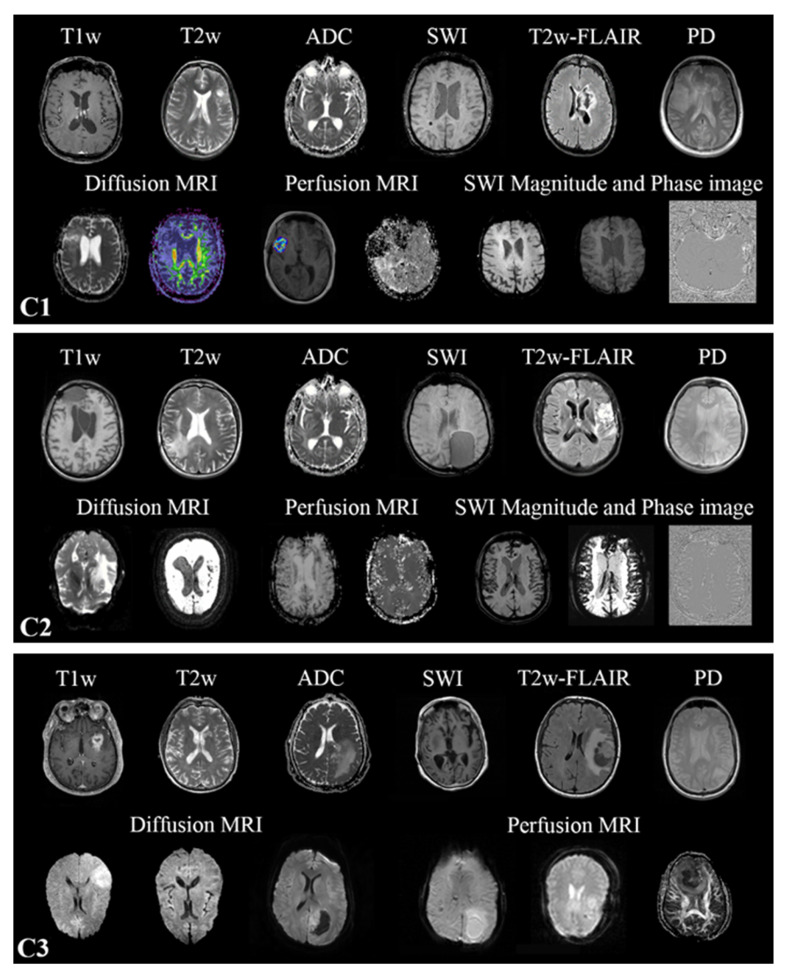
Sample images of the different MR images present in the three datasets C1–C3.

Multiparametric MRIs (mpMRI) were collected from multiple scanners in all three datasets, resulting in heterogeneous modalities and MR sequence protocols (Supplementary Table S3). Both conventional multislice (2D) scans acquired in the axial, sagittal, or coronal planes, as well as 3D scans, are available. The MR sequences found in the cohorts are the widely used sequences for brain tumor imaging [20] in clinical routines and trials [21,22,23]. All MR images discovered in the training cohort were utilized for training. However, one-vs-all DCNN classifiers were only trained for T1w, contrast-enhanced T1w (T1wce), T2w, T2w fluid-attenuated inversion recovery (FLAIR), apparent diffusion coefficient (ADC), and susceptibility-weighted imaging (SWI). It should be noted that no SWI scans were found in C3. The in-plane resolution ranged from 0.33 × 0.33 to 2 × 2 mm for C1, 0.45 × 0.45 to 1.40 × 1.40 mm for C2, and 0.45 × 0.45 to 1.14 × 1.14 mm for C3. The slice thickness of all MR scans ranged from 0.9 to 7.5 mm. Each MR image was manually labeled by human experts using an in-house interactive labeling tool. The “Series Description” (SD) and “Contrast/Bolus Agent” DICOM attributes were then extracted and compared to the derived labels to evaluate the consistency of the metadata. Sample images found in the training and testing cohorts are shown in Figure 1.

### 2.3. DCNNs Comparison Study

In the context of medical image retrieval and classification using DCNNs, three different DCNNs are present, i.e., ResNet-18 [15], Φ-Net [16], and DeepDicomSort [17]. Hence, a comparison study was performed where the architecture with the highest classification accuracy was adopted in the one-vs-all training approach. Both 2D and 3D ResNet-18 were considered. C1 was used for training, while C2 was for independent testing. C3 was not included in the comparison study as it did not contain all considered MR scans. The comparison study was only performed with the images belonging to one of the six classes considered, resulting in 11,246 MR from C1 (8997/80% for training, 2249/20% for validation) and 8326 MR from C2 for testing.

Brief descriptions of the exemplary models behind Φ-Net and DeepDicomSort are given. Visual Geometry Group (VGG) was introduced in 2014 by Simonyan and Zisserman in a paper titled “Very Deep Convolutional Networks for Large-Scale Image Recognition” [24]. The VGG network architecture is simple, formed with 3 × 3 convolutional layers stacked on top of each other as depth increases, pooling layers, and fully connected output layers. Residual Networks (ResNet) were introduced in 2015 to address the problem of degradation in network accuracy as the network depth increases [25]. In addition to the conventional DCNN architecture for classification purposes, which involves an alternating stack of convolutional, activation, and pooling layers, ResNet introduces skip-connections that allow the network to skip one or more layers. These skip connections fit the unmodified input from the previous layer to the next layer, preserving the original image signal through identity mapping. This approach helps to preserve the gradient norm and solve the degradation problem. A softmax layer is then added to the end layer to generate probabilistic predictions of the classes. Schematics of the ResNet and VGG architectures are shown in Supplementary Figure S1. Besides the dimensionality increase, no changes were applied to the 3D ResNet-18 architecture. Diagrams and explanations of the architectures of Φ-Net [15] and DeepDicomSort [17] are presented in the authors’ original papers.

#### 2.3.1. Data Preprocessing

Before training, different preprocessing steps were implemented. The preprocessing pipelines provided by the authors’ GitHub pages were used for the DCNNs trained with Φ-Net and DeepDicomSort. As for the 2D and 3D ResNet-18 DCNNs, magnetic field inhomogeneities of the T1w images were first corrected using the N4ITK algorithm [26]. After reorienting to a common orientation, in-plane cropping was performed to remove background voxels. Then, to account for resolution variability, all MR scans were resampled to a uniform pixel spacing of 2 × 2 mm^2^, and volumes were interpolated to a 2 mm slice thickness. Images were then cropped around the brain into a digital grid of 224 × 224 × 224. Padding was performed when the image shape was smaller than the target grid. Lastly, a z-score normalization of the brain voxels was applied to bring all MR images to the same intensity scale. The formula of the Z-score normalization is as follows:

(1) $$\frac{x-\mu }{\sigma }=z$$

where *x* is the voxel intensity, *μ* is the mean of the intensity distribution, and *σ* is the standard deviation.

#### 2.3.2. DCNN Training and Testing

The 2D and 3D ResNet-18 DCNNs were trained using the deep learning Python library PyTorch (1.7.1) [27]. A stochastic gradient descent optimizer with a momentum of 0.9 was used with a learning rate scheduler that started with 0.001 and decayed by 0.1 when the training loss did not decrease for three epochs. A categorical cross-entropy loss was considered as the loss function. A learning rate scheduler with a patience number of 3 was used. Early stoppage was performed when no improvement in the loss was observed for five successive epochs. The maximum number of epochs was 100. The batch size was 5 for the 3D ResNet and 50 for the 2D ResNet. The 2D ResNet-18 training included ten slices around the middle slice, extracted from the corresponding preprocessed MR scan acquisition plane. Φ-Net and DeepDicomSort were trained through the training code provided by the authors’ GitHub pages. All 4 DCNNs were finally tested on the independent C2, with the 2D DCNNs classifying an MR image as a class through a majority vote (25 slices for DeepDicomSort and 10 slices for the 2D ResNet-18). The 3D ResNet-18 models required 8–10 h of training time, while the 2D ResNet-18 models took 1.7–2 h. The models were trained on an Intel Xeon processor with 8 cores, 32 Gb of RAM, and an NVIDIA GeForce GTX 1060 graphics card (6 Gb). The average inference time was 0.15 s for a single 2D slice and 4.92 s for a 3D image.

### 2.4. MR-Class: One-vs-All DCNNs

MR-Class comprises multiple one-vs-all binary classifiers instead of a single multi-class classifier, as used in the comparison study. The rationale behind training multiple one-vs-all DCNNs is the open-set recognition problem and the difficulty of training a DCNN image classifier for every possible MR image. The architecture used for MR-Class was based on the DCNN that achieved the highest accuracy in the comparison study. The training was performed twice using scans from C1. The first training included all MR images available in the dataset, while the second was performed using only the image volumes of the six considered classes (the same images used in the comparison study during training). The latter was conducted to enable a fair comparison of the performance of the one-vs-all dual-class classifiers (MR-Class) against a multi-class DCNN classifier, both trained on the same number of images. For each binary classifier, the classes were defined as follows: class 1 included all images corresponding to the targeted class, while class 0 contained all remaining images in the dataset. A stratified dataset split (by class) of 80% for training and 20% for validation was used (as shown in Table 1).

#### 2.4.1. Training and Preprocessing

**Figure 2:**
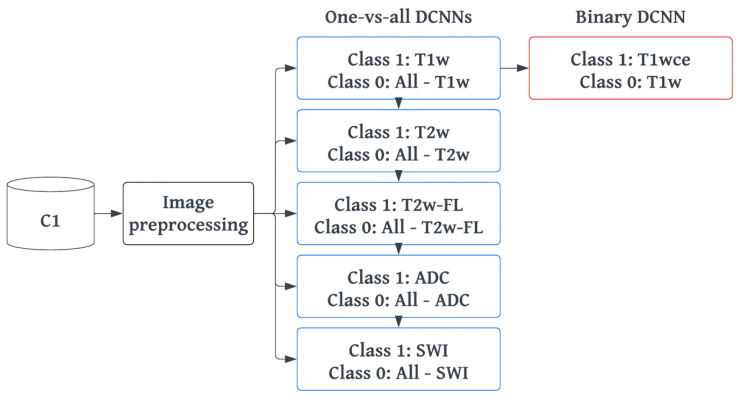
MR-Class training workflow. MR-Class comprises five one-vs-all DCNNs, one for each class, and the T1w-vs-T1wce binary DCNN. After MR image preprocessing, each DCNN was trained with an 80%/20% training/validation split, with class 1 representing the DCNNs’ target class and 0 for the rest. For the T1w-vs-T1wce DCNN, class 0 was assigned to T1w and 1 to T1wce. T2w-FL: T2w-FLAIR, T1wce: T1w contrast-enhanced.

The preprocessing and training approach employed for the 2D/3D ResNet-18 were also utilized for the one-vs-all DCNNs. However, additional steps were taken to address the imbalanced classes that arise from the one-vs-all classification design. Firstly, data augmentation was implemented using the TorchIO Python library [28]. This involved applying various transformations such as adding random Gaussian noise, blurring, performing random affine or elastic deformations, and adding random MR motion artifacts such as motion, ghosting, or spikes. Secondly, a weighted binary categorical cross-entropy loss was used, where the weights of a class were equal to the size of the largest class divided by the size of that specific class. Finally, the learning rate scheduler was adjusted to decay based on the targeted class training loss instead of the loss of both classes. Figure 2 provides an overview of the training workflow.

#### 2.4.2. Inference and Testing

**Figure 3:**
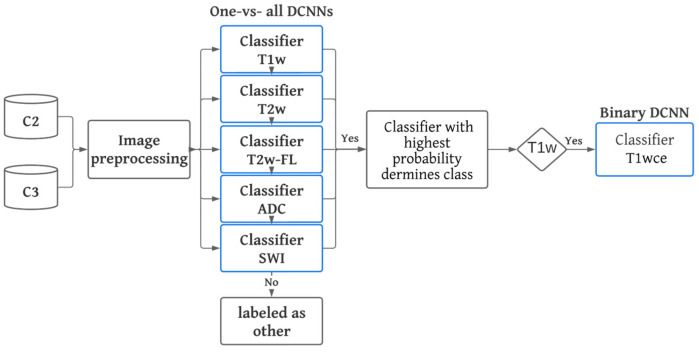
MR-Class inference workflow. C2 and C3 were used for testing. After preprocessing, MR images are passed to the 5 one-vs-all DCNN classifiers. A classification probability threshold of 0.95 was used. If none of the classifiers labels an image, it is rendered as other. If more than one classifier labels a specific image, then the image is labeled by the classifier with the highest probability.

C2 and C3 were utilized to conduct independent testing of MR-Class. During inference mode, the MR images were preprocessed using the same approach as during training and then passed to each DCNN classifier to infer the corresponding class. A classification probability threshold of 0.95 was employed, which was determined based on the distribution of the probabilities of correctly and incorrectly labeled images when C1 was inferred back to MR-Class. If an image is labeled by more than one classifier, the classifier with the highest probability determines the class. If none of the classifiers assigns a label to an image (i.e., assigned to class 0 by each classifier), it is deemed unclassifiable. For the 2D DCNNs, an MR scan is classified as a class based on the majority vote of 10 inferred slices extracted from around the middle slice of the corresponding MR acquisition plane. Figure 3 provides an overview of the inference workflow.

The classifications were compared to the ground truth labels, and accuracy was calculated as the number of correct predictions divided by the total number of images. A 95% confidence interval (CI) was determined using the Wilson interval method [29]. Classification sensitivity and specificity were also calculated to assess the performance of each classifier. Additionally, misclassified images were examined to determine the causes of misclassifications.

### 2.5. MR-Class Application: Progression-Free Survival Prediction Modeling

To demonstrate the applicability of MR-Class in MR-based radiomics applications, Cox proportional hazard models (CPHs) were trained with the T1wce MR sequences of cohort C2 to predict the patients’ progression-free survival (PFS) after performing a text-based curation using the DICOM SDs and a content-based curation using MR-Class [30]. PFS was calculated as the number of days between the beginning of the radiotherapy treatment and disease progression. Progression events were derived from the clinical follow-up reports. After performing a series of preprocessing steps on both curated datasets (DICOM SD-based and MR-Class-based curated datasets), radiomics features were calculated automatically from the gross tumor volume (GTV) segmentations extracted from the DICOM RT structure set and the original image, as well as from derived images (Wavelet and Laplacian of Gaussian filtering) from each dataset using Pyradiomics (v 3.0) [31]. The MR preprocessing diagram is shown in Supplementary Figure S3. The different feature classes and corresponding feature numbers can be seen in Supplementary Table S5. A Spearman rank-order correlation coefficient was next used on the total number of features to exclude redundant features (rs > 0.80). Three feature selection methods, including a univariate analysis under Cox proportional hazard (CPH) models (*p* < 0.05), a random forest (RF)-based method, and lasso regression, were applied separately on 1000 random subsamples of the text-based curated and MR-Class-curated T1wce datasets (10% left out) to identify features correlated to PFS. Significant features identified at least 950 times were selected, and survival analyses were conducted using CPH. Model performances were finally evaluated based on the resampled concordance index (C-I).

## 3. Results

### 3.1. Metadata Consistency

Between all three datasets, 2704 different DICOM SDs were found (an overview of the number of SDs found for each MR scan is shown in Supplementary Table S4). A total of 11.4%, 10.6%, and 10.7% of the SDs for C1, C2, and C3, respectively, had misleading or inconsistent entries, not allowing for the proper identification of the MR image class (Table 2).

### 3.2. DCNN Comparison Study

Table 3 summarizes the testing C2 MR scan classification accuracies of all four multi-class DCNN classifiers.

**Figure 4:**
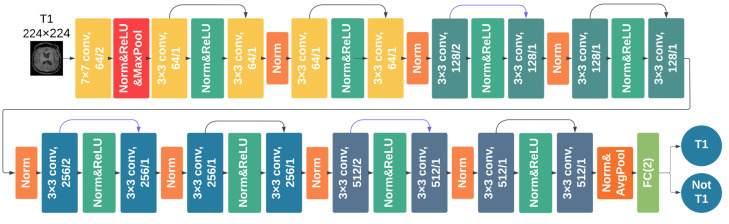
The one-vs-all ResNet-18 architecture. An alternating stack of convolutional activations and pooling layers. The skip connections (indicated by arrows) fit the unmodified input from the previous layer to the next, preserving the original image signal. FC (2) refers to a fully connected layer with two neurons as output, representing the sequence and the other possible sequences.

All classifiers achieved a high comparable accuracy, with the 2D ResNet-18 having the highest overall accuracy of 98.6%. The training took 18–20 h for the 3D DCNN (Φ-Net and 3D ResNet-18) and 8–10 h for the 2D DCNN (DeepDicomSort and 2D ResNet-18) on an Intel Xeon processor with 8 cores and 32 Gb of RAM and a graphics card NVIDIA GeForce GTX 1060 (6 Gb). The average inference time was 0.15 s for a single 2D slice and 4.92 s for a 3D image. Thus, the DCNN one-vs-all architecture implemented in MR-Class was that of the 2D ResNet-18 (a schematic representation is shown in Figure 4).

### 3.3. MR-Class: One-vs-All DCNNs

Table 4 summarizes the classification accuracies in the validation sets of all six DCNN classifiers on C1.

All six classifiers demonstrate high validation accuracies, with the lowest being 97.7% for the T1w-vs-T1wce and the highest being 99.7% for the SWI-vs-all, with a value of 99.6% for the ADC-vs-all tasks. Upon passing back the training set dataset I to MR-Class during inference mode, an accuracy of 97.4% [95% CI: 96.2, 98.4] was achieved, meaning that out of 20101 MR scans, MR-Class was unable to learn 519. In the multi-class versus multiple binary one-vs-all classification experiment, where only the image volumes of the six considered MR sequences were considered, the validation accuracy was comparable at 98.6% and 98.1%, respectively.

**Figure 5:**
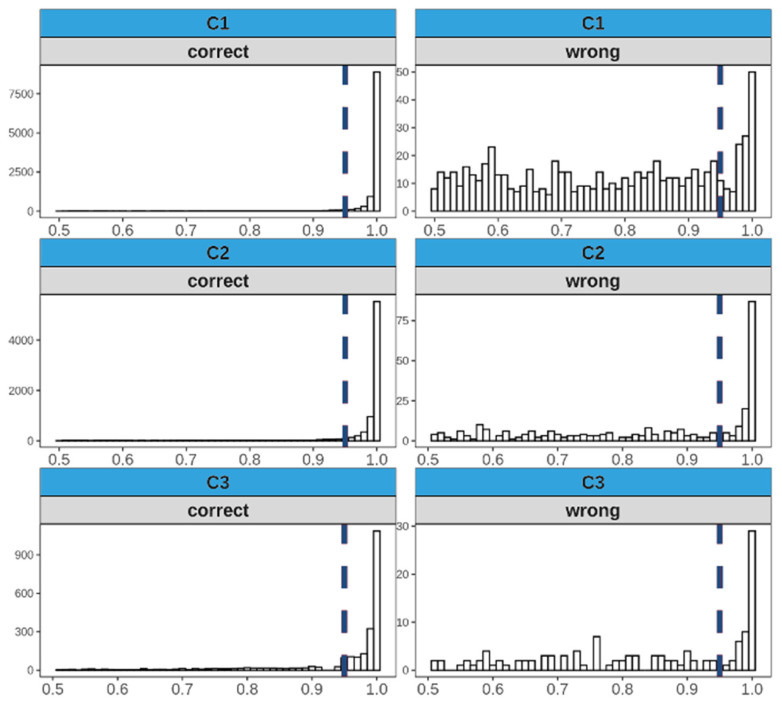
Distribution of the probabilities of correct and wrong labeled images for all three cohorts in the study when inferred to MR-Class. Based on the distributions of C1, a cutoff classification threshold probability of 0.95 was used. Histogram bin width = 0.01.

Figure 5 illustrates the distributions of the classification probabilities obtained by the MR-Class for all three cohorts. Based on C1, a probability cutoff threshold of 0.95 was set for testing MR-Class on C2 and C3.

**Figure 6:**
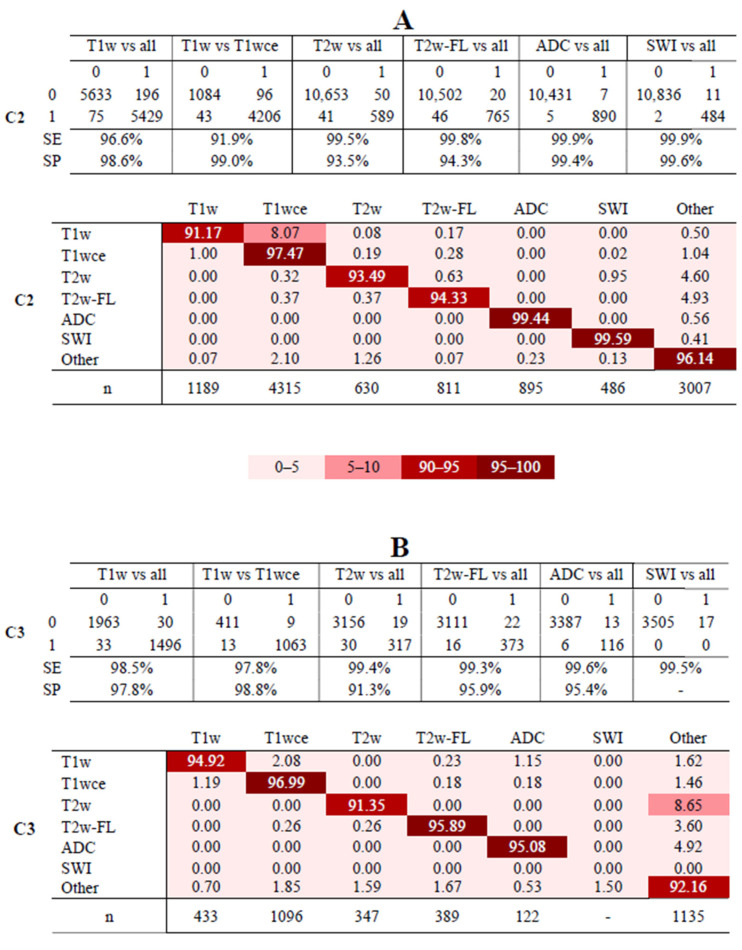
Confusion matrices of the six DCNNs for C2 (**A**) and C3 (**B**). The upper panels in (**A**,**B**) show the confusion matrices for datasets C2 and C3. The lower panels in (**A**,**B**) show MR-Class normalized confusion matrices for datasets C2 and C3, i.e., the percentages (%) of correct classification results per class. SE: sensitivity; SP: specificity. Class ‘Other’: when none of the DCNNs labels an image; *n*: number of scans per class, T2w-FL: T2w-FLAIR.

When tested against the independent C2 dataset, MR-Class achieved an accuracy of 96.7% [95% CI: 95.8, 97.3], with 424 out of 11333 images being misclassified. All DCNNs exhibited specificity ranging from 93.5% (T2w-vs-all) to 99.6% (SWI-vs-all). T1w-vs-T1wce and T1w-vs-all had the lowest sensitivity at 91.9% and 96.6%, respectively, while all other DCNNs had high sensitivity (>99%) (Figure 6A, upper panel). In the multi-class normalized confusion matrix (Figure 6A, lower panel), T1w classification was found to be the least reliable, with an accuracy of 91.17%. When tested against the independent C3 dataset, MR-Class achieved an accuracy of 94.4% [95% CI: 93.6, 96.1], with 196 out of 3522 scans being misclassified. T1w-vs-T1wce exhibited the lowest sensitivity at 97.4%, while all other DCNNs had a sensitivity greater than 98%. Specificity ranged from 91.3% (T2w-vs-all) to 98.8% (T1w-vs-T1wce) (Figure 6B, upper panel). In the multi-class confusion matrix (Figure 6B, lower panel), T2w classification was found to be the least reliable, with an accuracy of 91.35%, with 8.65% classified as “other”. The next section includes investigations on the misclassified images.

### 3.4. Analyses of Misclassified Images

**Figure 7:**
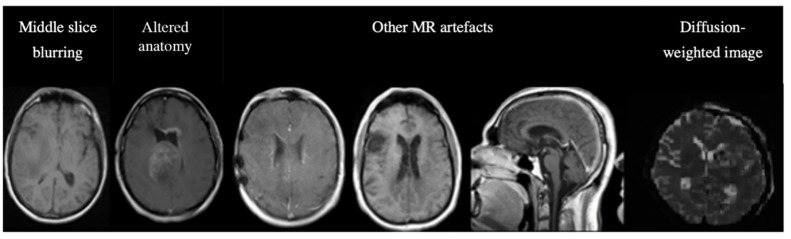
Examples of misclassified images. The first two images are examples of a misclassified MR, possibly due to blurry images (left) and alterations in expected anatomy (displaced ventricles, large tumor, right). The next three MR images show incorrect predictions due to different MR artifacts (shading, motion, aliasing). All of these images are falsely classified as “other”. The last image is a diffusion-weighted image (DWI), specifically a trace DWI, misclassified as T2w.

Of the 14,855 inferred images from C2 and C3, MR-Class classified 620 images incorrectly. The misclassifications can be sorted into different categories: MR artifact-middle slice blurring, MR artifacts-other, similar image content for different MR sequences (e.g., a T1w-FLAIR sequence instead of T2w), misclassified diffusion-weighted imaging (DWI) as T2w, and DICOM corrupted scans (sample images shown in Figure 7).

Upon manual evaluation, it was discovered that MR-Class frequently misclassified images (*n* = 122, 19.68%) when the ventricle architecture was altered, such as in cases where it was displaced by large tumors. This misclassification was investigated in detail by using 122 randomly selected correctly labeled images as a reference group. The ground truth volumes (GTVs) and brain were manually segmented, and the Euclidean distance between the CoM of the brain and the CoM of the tumor volume was calculated. A *t*-test was then performed between the reference and misclassified CoM distributions, which returned a *p*-value of 0.04. The median distance between CoMs was 46.15 voxels for the correctly labeled images and 66.31 for the misclassified images, indicating a statistical difference between the two groups. In other words, the further the GTV was from the ventricles, the less likely the image was to be misclassified. The frequencies of misclassification categories are presented in Table 5.

### 3.5. MR-Class Application: Progression-Free Survival Prediction Modeling

**Figure 8:**
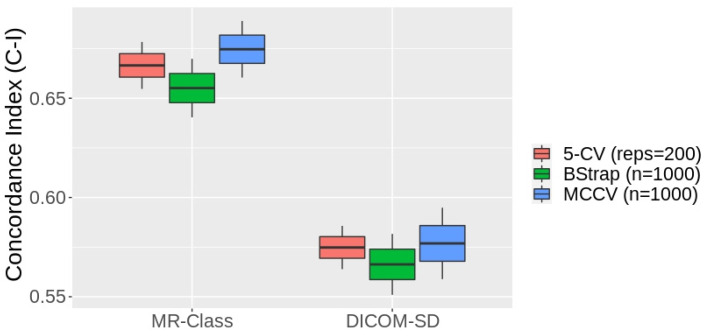
Box plots of the 1st–99th percentile C-Is attained by the MR-class and DICOM series description (SD) curated dataset models fitted by the respective signatures after three resampling approaches. MCCV: Monte Carlo cross-validation, BStrap: bootstrapping, CV: cross-validation.

Figure 8 shows the box plots of the 1st–99th percentiles resulting from the three resampling approaches following the fitting of the PFS CPH models by the radiomics signatures derived from the text-based and MR-Class-based curated datasets. Four and two significant features were identified from the text-based and MR-Class-based curated datasets, respectively. The average C-Is across the three different resampling approaches were 0.57 [0.55, 0.59] and 0.66 [0.64, 0.68] for the DICOM-SD and MR-Class models. The range represents the minimum and maximum C-I achieved. The DICOM SD-curated dataset included 7 misclassified T1w and 3 T2w sequences and excluded 10 T1wce images. The MR-Class-curated dataset excluded 4 misclassified T1wce images as they were labeled “other”.

## 4. Discussion

In this manuscript, we present an MRI image sequence classifier, MR-Class, which differentiates between T1w, contrast-enhanced T1w, T2w, T2w-FLAIR, ADC, and SWI while handling unknown classes. Testing was performed on two independent cohorts, where classification accuracies of 96.7% [95% CI: 95.8, 97.3] and 94.4% [95% CI: 93.6, 96.1] were observed. MR-Class consists of five one-vs-all DCNNs (one for each class), followed by a binary classifier for T1w images, to determine whether a contrast agent was administrated. This design enables MR-Class to handle unknown classes since each DCNN only classifies an image if it belongs to its respective class, and thus an image not labeled by any of the DCNNs is rendered as unknown. In order to compare the effectiveness of the multiple one-vs-all binary classifier approach with the basic multi-class classification method, we conducted a multi-class vs multiple dual-class classification experiment. The results showed that both methods achieved a similar classification accuracy for MR brain image classification, with the multi-class method achieving a 98.6% accuracy and the multiple one-vs-all method achieving a 98.1% accuracy. However, the latter approach has an advantage in that it can handle the open-set recognition problem often encountered when dealing with data from clinical cohorts, which can ultimately help reduce MRI study design times.

MR image DICOM series description (SD) entries usually follow the MR sequence protocol applied. However, they are MR model specific and are sometimes edited by clinical staff. We observed around 10% discrepancies in each cohort when the SD was compared to the manually derived labels. Typical SDs that do not allow for clear MR scan classifications are SDs with only the sequence name, e.g., spin echo (SE), or the scan direction, e.g., axial, which can stand for any MR sequence. Typographical errors and empty SD attributes were also observed.

Overall, high accuracies were obtained for all DCNNs in the comparison study. In conjunction with the high performance achieved in the literature on medical image classification [13,14,16,17], it is apparent that DCNNs can learn the intricacies behind different medical image modalities. In the DCNN architecture comparison study, 2D ResNet-18 had the best overall accuracy and thus was the architecture chosen for MR-Class. Furthermore, it was seen that the 2D DCNNs outperformed their 3D counterparts in MR sequence classification. MR scans correctly classified by the 2D DCNNs, while misclassified by the 3D DCNNs, are mainly conventional 2D axial, sagittal, or coronal scans with slice thicknesses ranging between 5 and 9 mm. Scans with a field of view that only encompassed the tumor area were misclassified by both 3D DCNNs (representative images can be seen in Supplementary Figure S2). It is important to note that no data augmentation was performed in the comparison study.

All six one-vs-all classifiers had high validation accuracies, with the lowest being 97.7% for the T1w-vs-T1wce. After inferring back the training cohort C1 to MR-Class, it was observed that 519 images could not be learned, out of which 336 belonged to class “other”, representing 3.8% of the other images used for training. This low error percentage demonstrates that MR-Class can learn to handle different sequences indirectly.

The testing of MR-Class against C2 and C3 yielded an average accuracy of 96.1%, where 620 images (4.2%) were classified incorrectly. Overall, T2w-vs-all had the worst performance, with a specificity of 93.5% and 91.3% in C2 and C3. This is mostly due to the presence of diffusion-weighted imaging (DWI) sequences (frequently encountered in the datasets), which are inherently a series of T2w sequences. Similarly, C3 included T1w-FLAIR images falsely misclassified as T1w or T2w-FLAIR. Thus, different sequences with similar content are prone to misclassification by MR-Class. A solution could be to train a subsequent classifier to distinguish between similar sequences, as performed for the T1wce images. The majority of misclassified images had severe blurring or other MR artifacts. A higher prevalence of misclassifications was observed in C3 compared to C2, and many of these misclassifications were false negatives, resulting in images being labeled as unclassifiable by MR-Class. This could be beneficial for radiomics models as any corrupted images would be automatically disregarded, and all images labeled as a specific class would have analogous content. Another subset of misclassified images showed tumor volumes overlapping with the ventricles, and statistical analysis confirmed that altered anatomy (specifically, ventricle displacement by large tumors) could be a possible reason for misclassification. However, further analysis is needed to assess the impact of surgery on alterations in overall anatomy, such as biopsy, partial resection, total resection, as well as the effect of treatments such as chemotherapy and radiotherapy on tumor patterns and contrast enhancement.

To improve the accuracy of MR-Class and reduce misclassifications, additional data including more diverse MR images should be considered. Additionally, for DWI images misclassified as T2w, a possible solution would be to train a subsequent classifier to distinguish between different MR image protocols, as was done for T1wce images. Another approach to reducing misclassification is to design a workflow that incorporates both text-based and content-based classification (MR-Class). In cases of mismatch between the two methods, manual intervention can be introduced to classify the image correctly. Misclassifications can be corrected through manual label adjustments, and upon the user’s acceptance, these images can be used to retrain the models using a transfer learning approach to improve MR-Class accuracy.

An essential step in building a radiomics application is to verify the input data labels before training the machine learning model, as inconsistent data can lead to the model drastically failing [32]. However, this was not performed while building the different survival models to demonstrate the applicability of MR-Class in MR-based radiomics applications. CPHs models were built with the T1wce MR sequences of cohort C2 to predict the patients’ PFS after performing a text-based curation using the DICOM SDs and a content-based curation using MR-Class. The MR-Class-curated model achieved an average C-I increase of 14.6%. This is mainly due to the content dissimilarity between the different images in the DICOM SD-curated dataset compared to the MR-Class-curated dataset.

MR-Class can facilitate the preparation of longitudinal studies for RT treatment assessment as MR data from the three cohorts include scans taken before, after, and throughout the delivery of the RT fractions, which resulted in different tumor volume masses between the different scans, as well as apparent radiation scaring in some of the MR images. Furthermore, the data cohort includes images taken directly after the surgical resection of the tumor, resulting in visible surgical holes and void tumor beds.

The 2D DCNNs in this study outperformed their 3D counterparts in classifying MR brain images. This was mostly due to the multiple conventional 2D multislice MR scans acquired in the axial, sagittal, or coronal plane in the three cohorts. However, the classification of MR sequences of a different entity, e.g., abdominal and pelvic MRI, might be more challenging and demand the intrinsic power of 3D DCNN. Due to the frequent presence of 2D images in MR datasets, the reconstruction of these low-resolution 2D slices to a high-resolution 3D MR might be a necessary preprocessing step before training. Nevertheless, the one-vs-all classification pipeline implemented in this study on brain MR images can be used for different anatomy sites and other medical image classification problems, for example, the classification of different body parts and organs.

## 5. Conclusions

MR-Class is a helpful tool for automating the classification of MR images, thus eliminating the need for manual sorting and saving time for researchers. The tool is particularly useful for studies involving large amounts of data and different naming schemes, as it classifies images based on their content rather than metadata, and can automatically disregard corrupted images. Future work includes expanding the tool to include additional modalities and sequences for different anatomical sites.

## Figures and Tables

**Table 1:** Number (%) of MR images from the training cohort, C1, considered for each one-vs-all DCNN classifier. T2w-FL: T2-FLAIR.

	Training		Validation	
DCNN Classifier	Targeted Class	Remaining Images	Targeted Class	Remaining Images
T1w-vs-all	3152 (15.7)	12,929 (64.3)	788 (3.9)	3232 (16.1)
T2w-vs-all	1576 (7.9)	14,505 (72.1)	394 (2.0)	3626 (18.0)
T2w-FL-vs-all	1535 (7.6)	14,546 (72.4)	384 (1.9)	3636 (18.1)
ADC-vs-all	1550 (7.7)	14,530 (72.3)	388 (1.9)	3633 (18.1)
SWI-vs-all	1183 (5.9)	14,898 (74.1)	296 (1.5)	3724 (18.5)

**Table 2:** Percentage of labeling errors for each class considered in the cohorts. T2w-FL: T2w-FLAIR.

	C1		C2		C3	
	*n*	% Error	*n*	% Error	*n*	% Error
T1w	2023	15.1	1189	11.2	433	13.4
T1wce	1917	13.9	4315	13.4	1096	9.9
T2w	1970	9.3	630	11.7	347	10.3
T2w-FL	1919	7.2	811	10.5	389	8.2
ADC	1938	7.6	895	8.4	122	5.5
SWI	1479	6.3	486	6.6	-	-
Other	8855	13.1	3007	7.3	1135	12.1
All	20,101	11.4	11,333	10.6	3522	10.7

**Table 3:** Classification accuracy of the different DCNN architectures in the study. T2w-FL: T2w-FLAIR.

	2D-ResNet	DeepDicomSort	Φ-Net	3D-ResNet
T1w	98.4	98.8	97.7	96.5
T1wce	97.4	95.2	97.5	96.2
T2w	98.1	97.2	96.6	97.1
T2w-FL	99.7	99.4	96.5	98.7
ADC	99.9	99.3	98.5	99.2
SWI	98.2	98.5	97.5	98.9
All	98.6	98.1	97.4	97.8

**Table 4:** Validation classification accuracies of all six binary DCNN classifiers on C1. T2wFL: T2w-FLAIR.

Classifier	Val Acc (%)	Classifier	Val Acc (%)
T1w-vs-all	99.1	T2wFL-vs-all	99.4
T1w-vs-T1wce	97.7	ADC-vs-all	99.6
T2w-vs-all	99.3	SWI-vs-all	99.7

**Table 5:** Frequency (*n*) and percentage (%) of the misclassified images.

Category	*n*	%
MR artifact-other	146	26.84
MR artifact-middle slice blurring	127	23.35
Tumor/GTV displacing ventricles	122	22.43
Similar content-different sequence	80	14.71
DWI as T2w	76	13.97
DICOM corrupted images	69	12.68

## Data Availability

MR-Class is available on our Github page, https://github.com/TRO-HIT/MR-Class, and is integrated into our big data curation tool for radiotherapy applications, PyCuRT [33] https://github.com/TRO-HIT/PyCRT. The public C3 used for testing can be downloaded at https://wiki.cancerimagingarchive.net/display/Public/TCGA-GBM. C1 and C2 are available from the corresponding authors on reasonable request.

## Supplementary Materials

The following supporting information can be downloaded at: https://www.mdpi.com/article/10.3390/cancers15061820/s1. Figure S1: An example of ResNet and VGG architectures with 18 and 16 layers and two output neurons; Figure S2: Images classified correctly and wrongly by 3D and 2D classifiers; Figure S3: T1wce preprocessing diagram applied before survival prediction modeling; Table S1: State-of-the-art methods specifications; Table S2: Patient demographics; Table S3: MR models found in the cohorts; Table S4: Number of series descriptions found for each class; Table S5: The number of shape, first- and second-order statistics derived per sequence and calculated on both the original and derived images.
