# Supplementary material for: MR-Class: A Python Tool for Brain MR Image Classification Utilizing One-vs-All DCNNs to Deal with the Open-Set Recognition Problem
Source: Cancers (Basel). 2023 Mar 17;15(6):1820. doi: 10.3390/cancers15061820 (PMC10046648; doi:10.3390/cancers15061820)
Supplement: Supplementary file 1 [file cancers-15-01820-s001.zip › cancers-2177751-supplementary.pdf]

cancers MDPI

Supplementary-Table S1. State of the art method specifications

**Paper/method**	Φ-Net Remedios et al. (2018) [16]	Ayyachamy et al. (2019) [15]	DeepDicomSort van der Voort et al. (2021) [17]	Qayyum et al. (2017) [14]
**Classes type**	5 MR image contrasts	23 body parts	8 MR image contrasts	24 body parts
**Scanners and sites**	5 MR scanners and 4 sites	MRI and CT	Experiment 1: 29 scanners from 17 sites 15 scanners from 8 sites Experiment 2: 23 scanners from 67 sites	MR, CT, PET, PT, OPT
**No. of images**	Training: 2137 Testing: 1281	Training: 21632 Validation: 9418 Testing: 5613	Experiment 1 – Training: 11065, Testing: 2369 Experiment 2 – Training: 7227 scans	Training: 5040 Testing: 2160
**Patients**	Healthy, traumatic brain injury, hypertension, multiple sclerosis, Alzheimer's disease	Chest, colon, esophagus, lung, liver, brain, prostate, head and neck	Experiment 1: Glioblastoma, brain neoplasia, other brain tumors Experiment 2: Alzheimer's disease	Lung, brain, liver, knee

**CNN architecture**	Borrows skip connections from 3D ResNet	Pre-trained ResNet-18	Inspired by the VGG network	DCNN
**Cascaded architecture For contrast agent enhancement**	yes	no	no	no
**Preprocessing**	Neck removal, resampling (2x2x2), intensity normalization 99th percentile	Resize to 256x256, 0-1 intensity normalized, tumor slice selection + 5 adjacent slices	NIfTI conversion, 3D dimension check, reorientation, resampling (256x256x256), slices extraction, 0-1 intensity normalization, no data augmentation	Resampling (256x256), color images converted to grayscale, no data augmentation
**Accuracy**	97.6%	92%	98.7%	99.7%
**Deals with the open set recognition problem**	No	No	No	No

Supplementary-Table S2. Patient demographics of the data cohorts considered in this study

	**Dataset I**	**Dataset II**	**Dataset III**
**Patients**	320	197	256
**Gender**			
Male	196	120	155
Female	124	77	101
**Age**			
<50	104	84	50
50-69	167	105	145
>=70	49	8	61
**Tumor grade**			
III	73	71	0
IV	247	126	255
**Radiation therapy**			
No	0	0	113
Yes	320	197	143
**Tumor resection**			
yes	240	25	0
No	10	172	0
NR	70	0	256

Supplementary-Table S3: MR scanner models found in the cohorts

Dataset	Manufacturer	Tesla	Model
**I**	Siemens	0.35	Open
		1	Allegra, Harmony
		1.5	Aera, Amira, Avanto, Espree, Sonata Symphony, Vision
		3	Prisma fit, Skyra, Trio, TrioTim,Verio
	Philips	1	Panorama
		1.5	Achieva, Ingenia, Intera,
		3	NT
	GE	1.5	Signa, Signa Excite-HDxt
**II**	Siemens	1	Harmony
		1.5	Avanto, Aera, Espree, Sonata, Symphony
		3	Prisma fit, Skyra, TrioTim,Verio
	Philips	1.5	Achieva, Ingenia, Intera
	GE	1.5	Optima MR450w, Signa HDxt
**III**	Siemens	1.5	Avanto, Espree, Sonata, Symphony
		3	Verio, Trio, TrioTrim
	Philips	0.5	T5
		1.5	Achieva, Intera
	GE	1.5	Signa, Signa Excite-HDx-HDxt
	Hitachi	0.3	Airis II

a)

b)

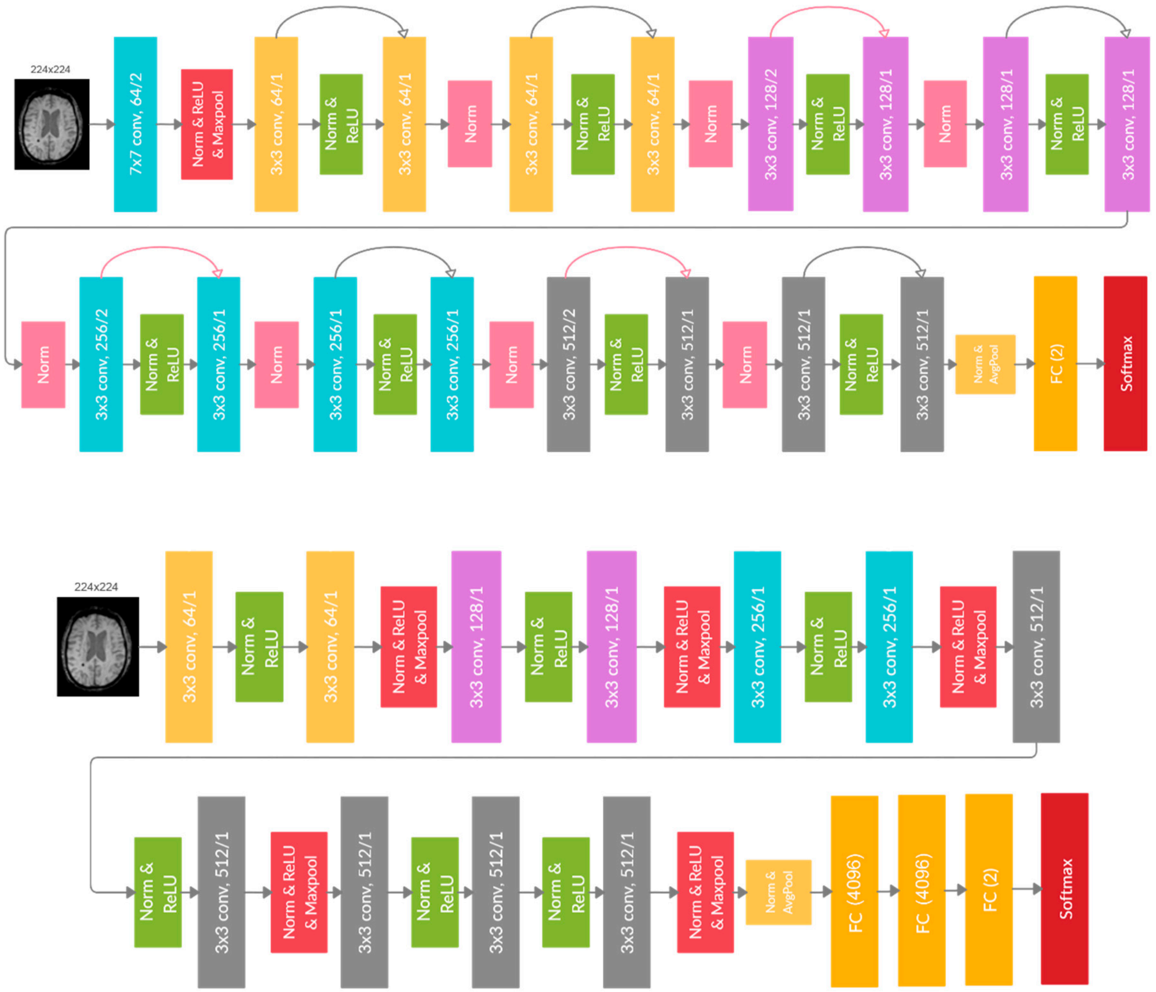

Supplementary-Figure S1: An example of ResNet and VGG architectures with 18 and 16 layers, and two output neurons. FC (2) represents a fully-connected layer with the two output neurons. a) ResNet-18 architecture. Stack of 3x3 convolutional layers, activations layers, and pooling layers. The skip connections, represented with arrows, fit the unmodified

input from the previous layer to the next layer, preserving the original image signal. A softmax layer is appended to the FC layer to produce probabilistic predictions of the classes. b) VGG-16 architecture. Stack of 3x3 convolutional layers, activations layers, and pooling layers, followed by FC output layers

Supplementary-Table S4: Nr. of series descriptions (SD) found for each class

	n			Nr of series description		
	I	II	III	I	II	III
Pre-CA T1w	2023	1189	433	1105	184	120
Post-CA T1w	1917	4315	1096	1060	464	158
T2w	1970	630	347	383	126	88
T2w-FLAIR	1919	811	389	223	122	63
ADC	1938	895	122	125	78	23
SWI	1479	486	-	61	8	-

A

B

C

D

E

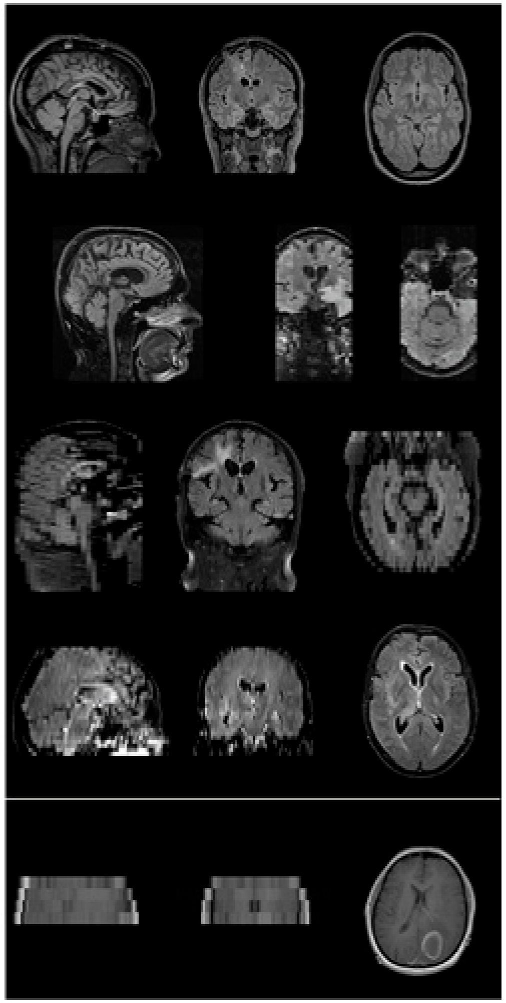

Supplementary-Figure S2: (A) a 3D reconstructed MR scan correctly classified by both the 2D and 3D CNNs. The remaining images are samples correctly classified by the 2D CNNs but misclassified by the 3D CNNs, specifically conventional 2D (B) axial, (C) sagittal (D) coronal acquired scans or (E) scans with field of views that only encompassed the tumor area

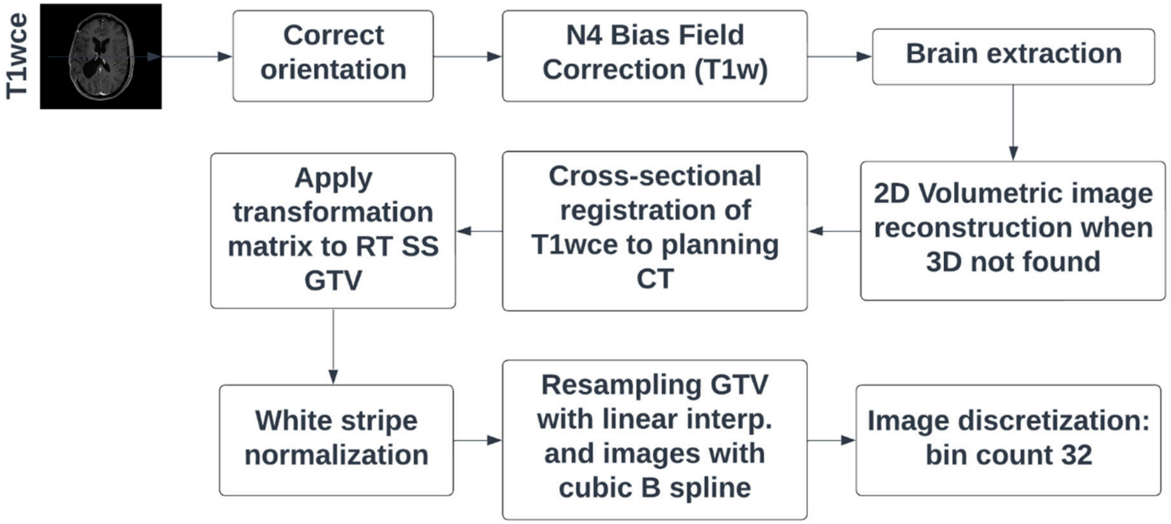

Supplementary-Figure S3: T1wce preprocessing diagram applied before survival prediction modeling.. RT SS GTV represents the gross tumor volume segmentation extracted from the DICOM RT structure set.

Supplementary-Table S5. The number of shape, first and second-order statistics derived per sequence and calculated on both the original and derived images.

Class	No. features
**First-order statistics**	19
**Shape-based (3D)**	16
**Second-order statistics**	
Gray Level Co-occurrence Matrix	24
Gray Level Run Length Matrix	16
Gray Level Size Zone Matrix	16
Neighbouring Gray Tone Difference Matrix	5
Gray Level Dependence Matrix	14
